# Alterations in Red Blood Cell Deformability during Storage: A Microfluidic Approach

**DOI:** 10.1155/2014/764268

**Published:** 2014-09-10

**Authors:** Judith C. A. Cluitmans, Venkatachalam Chokkalingam, Arno M. Janssen, Roland Brock, Wilhelm T. S. Huck, Giel J. C. G. M. Bosman

**Affiliations:** ^1^Department of Biochemistry, Radboud Institute for Molecular Life Sciences, Radboud University Medical Centre, Geert Grooteplein 28, 6525 GA Nijmegen, The Netherlands; ^2^Department of Physical Organic Chemistry, Radboud University Nijmegen, Institute for Molecules and Materials, Heyendaalseweg 135, 6525 AJ Nijmegen, The Netherlands; ^3^Department of Neurology, Radboud University Medical Centre, Donders Institute for Brain, Cognition and Behaviour, Reinier Postlaan 4, 6525 CG Nijmegen, The Netherlands

## Abstract

Red blood cells (RBCs) undergo extensive deformation when travelling through the microcapillaries. Deformability, the combined result of properties of the membrane-cytoskeleton complex, the surface area-to-volume ratio, and the hemoglobin content, is a critical determinant of capillary blood flow. During blood bank storage and in many pathophysiological conditions, RBC morphology changes, which has been suggested to be associated with decreased deformability and removal of RBC. While various techniques provide information on the rheological properties of stored RBCs, their clinical significance is controversial. We developed a microfluidic approach for evaluating RBC deformability in a physiologically meaningful and clinically significant manner. Unlike other techniques, our method enables a high-throughput determination of changes in deformation capacity to provide statistically significant data, while providing morphological information at the single-cell level. Our data show that, under conditions that closely mimic capillary dimensions and flow, the capacity to deform and the capacity to relax are not affected during storage in the blood bank. Our data also show that altered cell morphology by itself does not necessarily affect deformability.

## 1. Introduction

During its life span of approximately 120 days, a red blood cell (RBC) makes about 160,000 round-trips between the lungs and the tissues [1]. Its high flexibility enables passage through microcapillaries, some of which have a diameter smaller than the RBCs themselves, thereby ensuring transport of oxygen and carbon dioxide throughout the body. Within the microcapillaries, RBCs repeatedly change from a discocyte shape to an axisymmetric bullet-like shape, depending on the flow properties and microcapillary diameter, and back to a discocyte shape within the venules [2]. This extensive deformation and relaxation, which is a critical parameter of the blood flow, are the combined result of the elastic properties of the membrane-cytoskeleton complex, the surface area-to-volume ratio, and the viscosity as determined by the hemoglobin content [3].

During storage in the blood bank, RBCs undergo several structural and functional changes [4]. Some of these changes are likely to induce the clearance of up to 30% of the transfused RBCs from the circulation within 24 hours after transfusion [5]. One possible explanation for this disappearance may be a critical decrease in deformability during storage [6, 7], which could lead to a decrease in the capacity to pass the narrow interendothelial slits in the spleen, and a concomitant increase in susceptibility to phagocytosis [8]. Also, various RBC-centered pathophysiological conditions that are associated with microvascular disorders are associated with altered RBC deformability [9–11]. However, little is known on the underlying molecular mechanisms.

A number of techniques have been developed to quantify RBC deformability. The available methods can be divided into two categories: single-cell techniques, such as micropipette aspiration and optical tweezers, and measurement on whole blood or diluted RBC suspensions, such as filtration and ektacytometry [12]. Single-cell techniques, while precise, make it difficult to obtain statistically significant data, whereas the RBC suspension techniques impose flow geometries that are far from those actually experienced by RBCs in human capillaries. Also, these techniques are only able to measure flow behavior of populations or endpoint results. Because of these limitations, these techniques have yielded confusing and sometimes even conflicting results [3, 13–15]. The perfusion of RBCs through microcapillaries provides an alternative and more direct means to address RBC deformability [16, 17]. Most work in this area so far has focused on understanding the physical rather than the biological aspects of RBC deformability [18–21]. Therefore, we developed a microfluidic system for the study of red blood cell flow behavior in a microcirculation-mimicking network. In addition to assessing deformation in microchannels with dimensions similar to those of microcapillaries, we also monitored relaxation to the discocyte shape. Using high-temporal resolution imaging, we could record time-lapse videos of RBCs in the narrow channels and the relaxation zones. Deformation and relaxation were analyzed by automated image processing that provides statistically significant data. This approach enables us to address the effect of heterogeneity within the RBC population. The visual aspect of our approach makes it possible to relate the relaxed morphology to the deformed state on the single-cell level. These characteristics make our microfluidic system useful not only for identification of the molecular determinants of RBC deformability, but also for evaluation of the effect of RBC pathology (abnormal hemoglobins, membranopathies, and enzyme deficiencies) on the microcirculation and of the consequences of pathological conditions such as systemic inflammation or cardiovascular disease on RBC deformability.

After establishing the conditions to measure RBC deformability in a physiologically meaningful and clinically significant manner, we studied the association between blood bank storage and deformability, since this could serve as a possible explanation for the considerable loss of RBCs shortly after transfusion and for the mostly unknown relationship between blood bank quality parameters and RBC function and behavior after transfusion [4, 5].

## 2. Materials and Methods

### 2.1. Ethics Statement

All procedures followed were in accordance with the ethical standards of the committee on human experimentation (CMO NL number: 45934.091.13). Written informed consent was obtained from all blood donors participating in this study.

### 2.2. RBC Sample Preparation

Experiments were performed with RBCs from five transfusion units that had been collected, processed, and stored following standard Dutch blood bank procedures, including removal of buffy coat, leukoreduction, and storage in saline-adenine-glucose-mannitol. RBCs were washed to remove medium, plasma, and vesicles using Ringer solution (125 mmol/L NaCl, 5 mmol/L KCl, 1 mmol/L MgSO_4_, 2.5 mmol/L CaCl_2_, 5 mmol/L glucose, 32 mmol/L HEPES/NaOH, and pH 7.4) by repeated centrifugation (5 min, 1550 g). Fresh RBCs were isolated from 5 mL whole blood (EDTA), donated by healthy volunteers. RBC fractionation according to cell density was performed using a discontinuous Percoll gradient consisting of six layers ranging from 40% Percoll to 80% Percoll as described previously [22]. The RBCs were combined into four fractions: fraction 1, 61% Percoll + 64.5% Percoll; fraction 2, 67.5% Percoll; fraction 3, 71% Percoll; and fraction 4, 80% Percoll. All deformability analyses were performed in Ringer containing 1% bovine serum albumin (BSA, Sigma-Aldrich, St. Louis, MO, USA).

### 2.3. Treatment of RBCs

In order to investigate the contribution of the various cell components to the deformability in our microfluidic system, RBCs were incubated for 20 min at room temperature with 0.05% glutaraldehyde (GA), obtained by dilution of a 25% GA-stock solution (Sigma-Aldrich, St. Louis, MO, USA) with Ringer. Alternatively, treatment with lysophosphatidylcholine (LPC) (Sigma-Aldrich, St. Louis, MO, USA) consisted of incubation at room temperature at 10% hematocrit for five minutes with 5 to 10 *μ*M LPC in Ringer.

### 2.4. Phosphatidylserine (PS) Measurements

The percentage of PS-exposing RBCs, a physiological indication for the possibly damaging effect of the stress that the RBCs may have experienced, was determined as described before [23]. In short, RBCs were incubated for 30 min at room temperature in the dark in Ringer with Annexin-V-FLUOS (1 : 25, Roche, Basel, Switzerland) to label PS-exposing cells. The RBCs were analyzed with a flow cytometer (FACScan, Becton Dickinson, Franklin Lakes, NJ), using its accompanying software (CELLQUEST, Becton Dickinson). The data were analyzed with Summit 4.3. Results are expressed as percentages of Annexin V-positive RBCs.

### 2.5. Microchannel Fabrication

**Figure 1:**
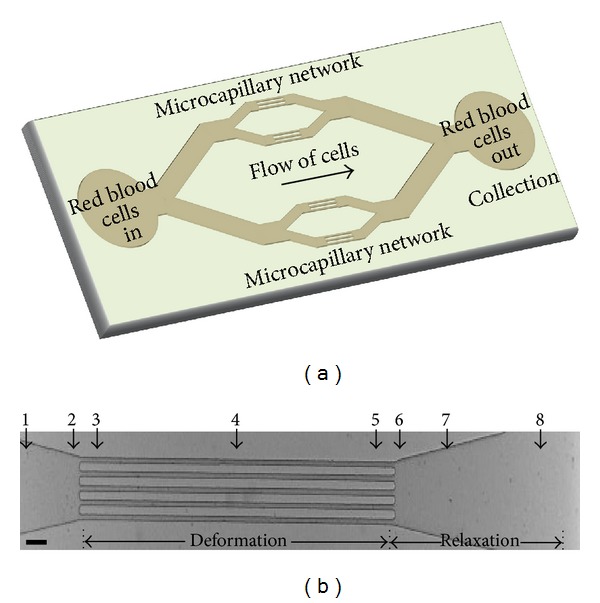
Microcapillary network design. (a) Schematic of the whole microfluidic device. (b) Microchannel region selected to image RBC deformation and relaxation. Numbers indicate the different spots/regions of interest within the channel that were used for the analysis of RBC shape change. Scalebar: 50 *μ*m.

Soft lithographic techniques were used to fabricate a microcapillary network of 10 *μ*m deep channels (Figure 1; [24, 25]). The microfluidic device was molded against an SU-8 photoresist structure on a silicon wafer using a commercially available poly(dimethylsiloxane) (PDMS) silicone elastomer (Sylgard 184, Dow Corning, USA). The surface of the Sylgard 184 devices was OH-terminated by exposure to plasma (Diener electronic, Germany) and was sealed with another plasma-treated glass cover slide to yield closed microchannels. The liquids were dispensed from plastic syringes (Becton Dickinson), which were connected to the microfluidic device by polytetrafluoroethylene tubing (Novodirect, Kehl, Germany).

### 2.6. Experimental Setup and Data Analysis

For monitoring RBCs in microfluidic flow, cells were diluted to a concentration corresponding to a hematocrit of 2%. The cells were perfused through the microfluidic channels with computer-controlled syringe pumps (neMESYS, Cetoni, Germany) to enable accurate, constant volumetric flow rates. Images of the field-of-view were recorded (with up to 1250 frames per second) for quantitative image processing. Flow of cells was observed through a 100X oil immersion objective (Olympus UPLFLN 100X, N.A. 1.30) using an optical microscope (IX71, Olympus B.V., The Netherlands), equipped with a high speed CMOS camera (Phantom high speed camera, Vision Research, UK). High quality snapshots of RBCs were obtained using a short exposure time down to 10 *μ*s. In each run, a sequence of images of approximately 20,000 was recorded. The videos were manually preprocessed with* Image J* to divide them into separate video files for the various regions of interest (ROIs; Figure 1(b)). Hereafter, RBC detection and DI calculation were performed fully automatically with a custom-written MATLAB code. The grey scale video files from* Image J* are used as input, and the output consists of DI values for all detected RBCs in the input files. The automatic RBC detection and DI calculation are split up in seven steps. Each of the following steps was performed for each video file individually. First, a subset of frames (20 percent) was taken for analysis. Second, background filtering was performed on the subset of frames by subtracting an averaged image, based on multiple frames. This procedure eliminates static objects, such as the microfluidic channels. Third, the grey scale frame images were transformed into binary black-white images, based on a preset grey intensity threshold. Fourth, RBCs were detected per frame as white pixel clusters in the black-white images. Fifth, pixel clusters, representing aggregated RBCs and incomplete RBCs on the edge of the field, were excluded automatically from the dataset. Sixth, the length, width, and area were automatically determined for each included RBC (white pixel cluster). Seventh, the deformation index (DI), defined as the ratio between the sides (DI = length/width), was calculated for each individual RBC.

### 2.7. Statistical Analysis

Data analysis was performed using PASW Statistics 18 (IBM, New York, USA). All DI values are mean values, calculated from at least 800 events per sample (represented by 200–300 cells). A *P* value less than 0.05 was considered statistically significant. Differences between the mean values were evaluated using one-way ANOVA, followed by least significant difference correction for multiple comparisons. In a first approximation, it was assumed that repeated measurements did not influence the results. Linear regression was applied to verify that DI remained constant within the channels. In all cases, the *t*-test for individual regression coefficients showed that the slope did not contribute significantly to the linear model and hence could be omitted, leaving a constant value.

## 3. Results

We aimed to measure RBC flexibility in a clinical context and under physiologically relevant conditions. Therefore, next to assessing the capacity of RBCs to deform in narrow channels, we also addressed relaxation upon exit from these channels. Furthermore, given the heterogeneity in RBC populations from one donor [26, 27], we also aimed to obtain data at a single-cell level. In order to meet these requirements, we established a microfluidic system with high-speed image acquisition and quantitative image analysis (Figure 1). The parallel array of microfluidic channels not only enhances throughput, but also enables collection of sufficient numbers of RBCs for biochemical analysis.

To establish the physiologically relevant conditions for quantitatively assessing erythrocyte deformation in flow through narrow microchannels, we examined the effect of length and width of the channels and the flow velocities along these channels. A channel length of 1000 *μ*m was selected because at that length all RBCs exhibited the axisymmetric bullet-like shape found in themicrocirculation *in vivo*. The deformation channels open into a wider region to allow the cells to relax back to their original shape (Figure 1). Analysis of the flexibility was performed at the exit of the deformation channels, enabling a simultaneous detection of the deformation and relaxation of a single RBC.

**Figure 2:**
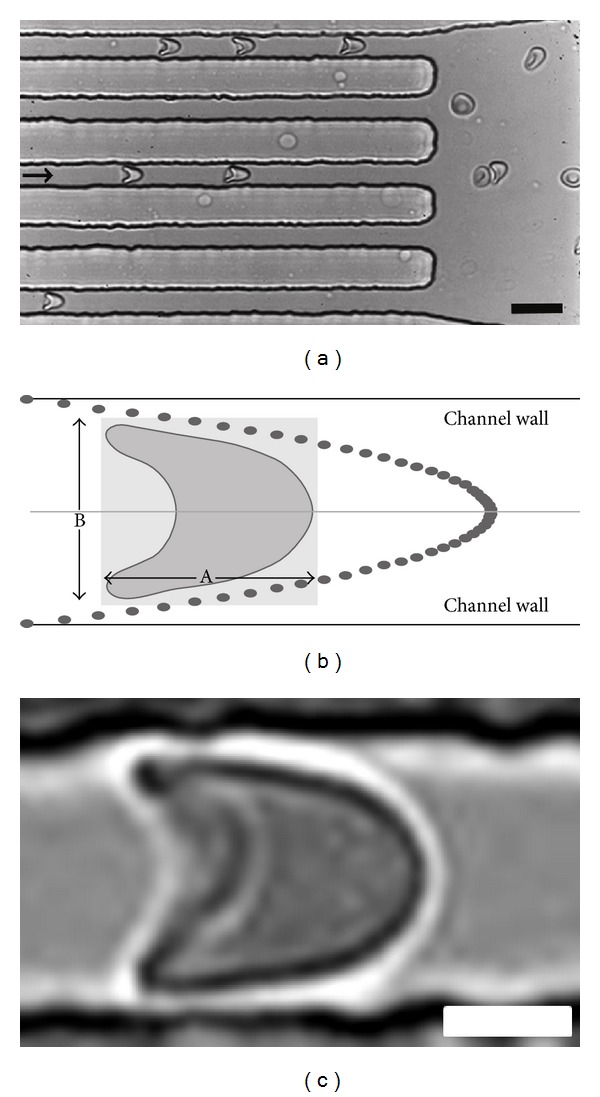
Flow of RBCs in microchannels. (a) RBCs flowing inside and just outside microchannels. The arrow indicates the direction of the flow. Scalebar: 15 *μ*m. (b) Deformation of the cell is determined in a virtual box with length (A) and width (B) and is expressed by the deformation index (DI), that is, the ratio between the length and width (A/B). Dots indicate the parabolic flow profile within the microfluidic channels. (c) Deformed single RBC flowing within the 7 *μ*m channel showing a bullet-like shape. Scalebar: 3 *μ*m.

**Figure 3:**
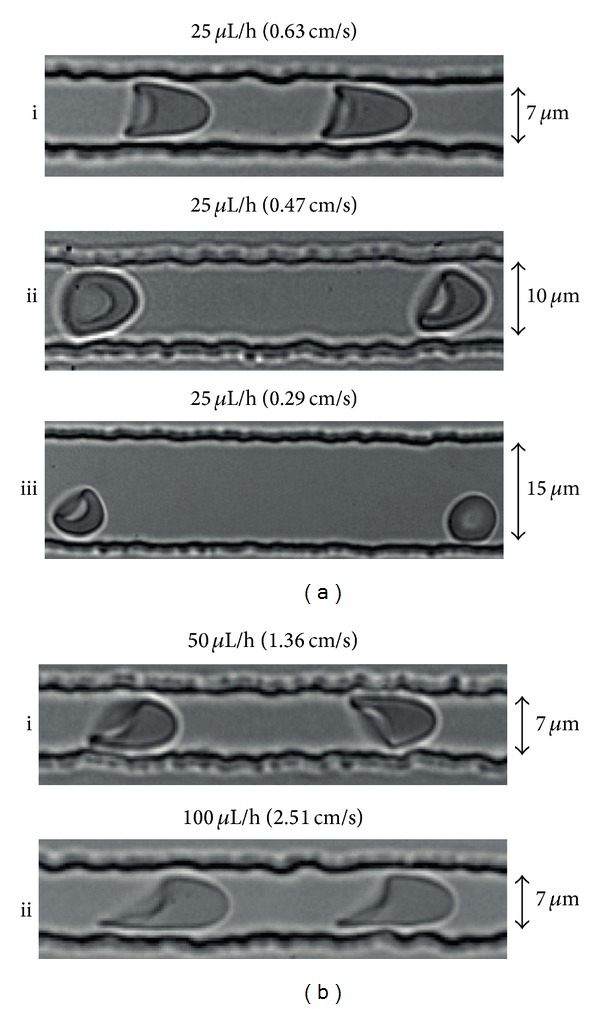
Deformation of RBCs exposed to various flow parameters. (a) Channels with widths of  7, 10, and 15 *μ*m and a syringe pump flow rate of 25 *μ*L/h, resulting in a flow velocity in the narrow channels of 0.63 *μ*L/h, 0.47 *μ*L/h, and 0.29 *μ*L/h, respectively, and in different cell shapes. (b) Behavior of cells at higher flow velocities of 1.36 cm/s and 2.51 cm/s (corresponding to pump flow rates of 50 *μ*L/h and 100 *μ*L/h).

The RBCs exhibited a bullet-like shape in the deformation part (Figure 2; see also video S1 in the supporting information, available online at http://dx.doi.org/10.1155/2014/764268). This RBC deformation in the narrow channels is expressed as the deformation index (DI), which is defined as the ratio between the length (A) and width (B) of the RBC during flow within this channel (Figure 2). In order to mimic flow conditions* in vivo*, the width of the microchannels was varied. In the smallest channel with a width of 7 *μ*m, at a flow rate of 25 *μ*L/h and a flow velocity of 0.63 cm/s in the narrow part of the channel, all RBCs showed the typical axisymmetric bullet-like shape (Figure 3) as described* in vivo*[2]. Under these conditions, the flow profile was parabolic and mimicked the normal blood flow in the human circulatory system [2, 28]. The corresponding shear stress (*τ*) was calculated using the Weissenberg-Rabinowitsch equation: *τ* = 6*Qμ*/*nWH* ^2^ (where *Q* is the total flow rate in *μ*L/h, *μ* is the viscosity in Pa*·*sec, *W* is the width of the channels in *μ*m, *H* is the height of the channels in *μ*m, and *n* is the number of channels). This rheometric equation gives the unique relation between the channel wall shear stress and the total flow rate for non-Newtonian fluids within rectangular channels [29, 30]. The shear stress was calculated to be 2.98 Pa, which is in the same range of that in human microcapillaries [31, 32]. In channels wider than 7 *μ*m, most of the RBCs exhibited tumbling behaviors at a flow rate of 25 *μ*L/h (Figure 3).

Deformation of RBCs was also analyzed at flow rates of 50 and 100 *μ*L/h, resulting in flow velocities of 1.36 cm/s and 2.51 cm/s in the deformation zone, in 7 *μ*m deformation channels. Asymmetric shape deformations were observed in channels for flow velocities above 0.63 cm/sec (Figure 3), resulting from out-of-axis cell position. The behavior of RBCs in 7 *μ*m channels at a flow velocity of 0.63 cm/s closely resembled that in the microcirculation* in vivo*[2, 31, 32]. Therefore, these conditions were used in further analyses.

RBCs were analyzed in Ringer solution instead of autologous plasma for ease of RBC preparation and because plasma has a higher risk of clogging the channels; this did not lead to significant differences in the deformation index and relaxation (DI 1.63 ± 0.29 in Ringer versus 1.61 ± 0.10 in plasma).

The microfluidic device enables the collection of RBCs for off-line analyses after passage through the device. The parallel array of microfluidic channels and a flow rate of 25 *μ*L/h enable the collection of 10 million cells within 20–30 minutes for off-chip analysis. PS exposure on the outer membrane is a stress signal which facilitates recognition and removal by macrophages [23, 33]. We used the possibility to collect RBCs after passage through the microchannels in order to evaluate any putative artefacts that would hamper the translation of our* in vitro* data to RBC behavior* in vivo*. Cells were collected at the end of the device and stained with Annexin-V. Flow cytometric analysis showed that any mechanical stress within the microfluidic device did not cause PS exposure (2.46 ± 0.01% PS-exposing cells before the passage through channels, compared to 2.24 ± 0.01% after passage). This observation supports the suitability of our setup in mimicking the conditions experienced* in vivo*.

**Figure 4:**
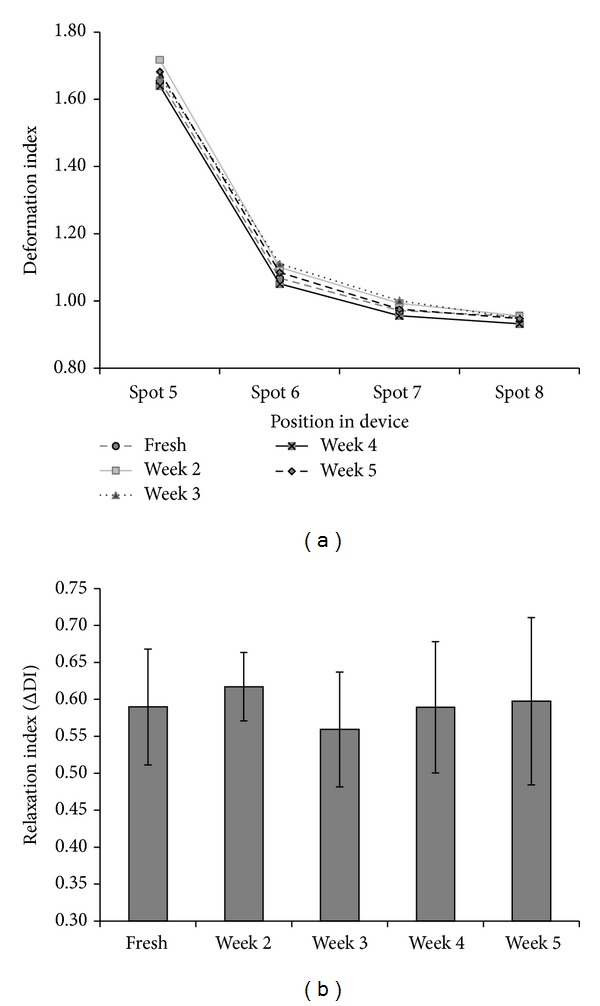
Deformation and relaxation of RBCs stored for different periods of time. (a) Deformation index of RBCs within the deformation channels (spot 5 in Figure 1) and relaxation regions (spots 6, 7, and 8 in Figure 1). For reasons of legibility the standard deviation, which varied between 15 and 20%, is not shown. There was no significant change in DI with storage time (*P* < 0.05). (b) Relaxation of RBCs stored under blood bank conditions for different periods of time. The relaxation capacity of RBCs is expressed as the ΔDI, that is, the difference in DI in the deformation channel (spot 5 in Figure 1) and in the relaxation region (spot 6 in Figure 1). All data are the mean of five different blood bags.

**Figure 5:**
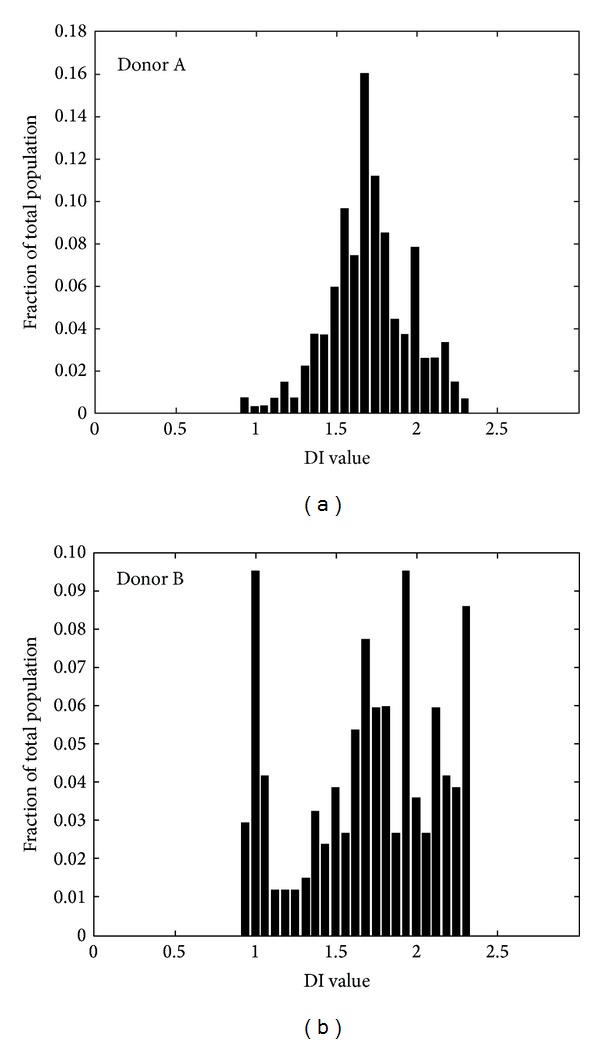
Donor heterogeneity in RBC deformability of RBCs. Given is the frequency distribution of DI for the RBCs from freshly drawn blood from two different donors. Per sample 250–350 events are measured.

Using our microfluidics setup, we investigated the effect of storage time in blood bank units on deformability and relaxation in a microcapillary-simulating environment. After transfusion, up to 30% of the transfused RBCs are cleared from the circulation within 24 hours, and changes in deformation may contribute to this phenomenon [5, 6]. RBCs of different storage times were perfused through the microfluidic device. In order to compare deformability, the DI of the cells was calculated at different locations within the device, in the microchannels as well as in the relaxation zone. Our data showed that storage in blood bank conditions for up to five weeks (the maximal storage time in the Netherlands) did not lead to a significant decrease in the capacity to deform to a bullet shape or to relax to a discoid shape afterwards (Figure 4). There were no indications for loss of RBCs or hemolysis in any of the samples, indicating the absence of a selection bias. The relatively large standard deviation suggests a large variation in deformation capacity of RBCs from different donors. This was confirmed by frequency distribution analysis of the DI values (Figure 5).

Since the standard deviation within the individual samples also suggested a heterogeneous deformation capacity, we postulated that this might be associated with heterogeneity in cell morphology. It is known that storage is associated with both a decrease in RBC density and an increase in abnormal cell morphology [4, 34]. Therefore, we measured the deformation behavior of RBCs of various densities as obtained by Percoll density separation. Although the stored RBCs in the various Percoll fractions had distinct morphologies, with the more aberrant cell shapes in the more dense fractions as has also been described by others [34], the mean DI of the RBCs did not differ significantly between the fractions (data not shown). These data indicate that a nondiscocyte morphology is not necessarily associated with decreased deformability as measured in capillary-mimicking conditions.

**Figure 6:**
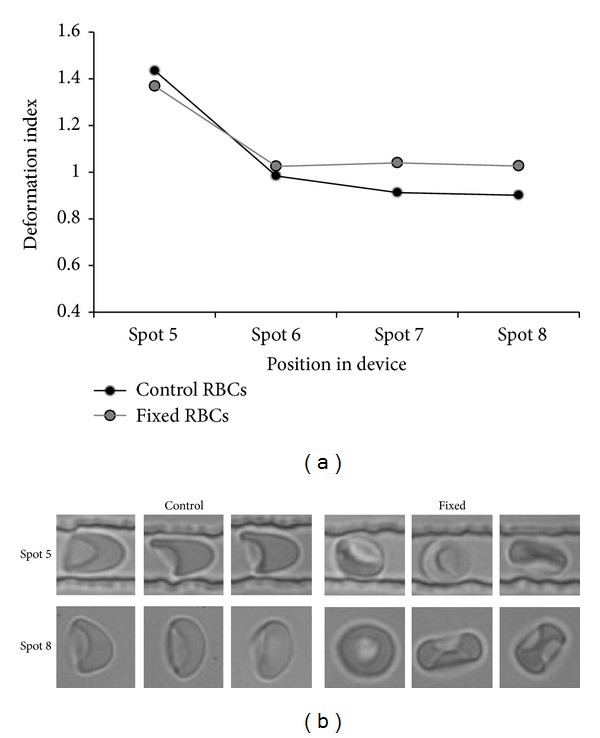
The effect of treatment with glutaraldehyde on deformability and relaxation capacity. (a) The deformation capacity of control and glutaraldehyde- (GA-) treated RBC samples as measured by the DI. For reasons of legibility the standard deviation, which varied around 15%, is not shown. At all spots, the DI of the GA-treated RBCs was significantly different from that of control RBCs (*P* < 0.01); (b) typical cell shapes in control and GA-treated samples in region of interest 5 (deformation) and region of interest 8 (relaxation).

Deformability is determined by the elastic properties of the membrane-cytoskeleton complex, the surface area-to-volume ratio, and the cellular viscosity, mostly determined by the hemoglobin concentration. In order to identify the contribution of these factors, we analyzed the deformation of RBCs that had been altered with glutaraldehyde or with LPC. Treatment with the fixative glutaraldehyde, which diminishes the elasticity of the membrane-cytoskeleton complex, has been described to impair RBC filterability [35], whereas incubation with LPC, which leads to a decrease in the surface-to-volume ratio, has been described to induce an increase in splenic retention* ex vivo* [36]. Most glutaraldehyde-treated RBCs kept their fixed morphology in the narrow channels, leading to a deformation index that was significantly (*P* < 0.05) different from that of untreated cells (Figure 6). Control cells relaxed back to their discocyte shape within maximally 40 ms after exiting from the narrow channels into the wider region of the device (video S1 in the supporting information), while the glutaraldehyde-treated RBCs kept the same fixed morphology also in this relaxation zone. The difference in DI for the glutaraldehyde-treated RBCs measured in the narrow channel and in the relaxation region is probably caused by the tumbling behavior the cells exert in the relaxation area, where there are no physical restrictions. Treatment of RBCs with LPC did not show any significant effects on deformation index or relaxation (data not shown), indicating that changes in membrane stiffness as induced by glutaraldehyde fixation affect the deformation index in the 7 um width microcapillaries, while moderate changes in the surface-to-volume ratio do not.

## 4. Discussion

Here we demonstrate that high temporal resolution imaging of RBCs at the exit zone of narrow channels within a microfluidic device provides comprehensive information on their capacity to deform and subsequently relax to a discoid shape. Using single-cell imaging, high throughput for statistically significant data was obtained by virtue of fully automated image processing. Furthermore, by parallelization of several microchannels, sufficient throughput can be generated for off-line analysis, for example, using flow cytometry. In addition, the visual aspect of this method makes it possible to study subpopulations within one sample, making it an attractive method to study red cell pathologies. By analyzing cells at the exit zone of the narrow channels, a simultaneous detection of a deformed and relaxing RBC can be performed, thereby correlating deformation behavior to specific morphology of RBCs in rest.

It has been postulated that the rapid clearance of up to 30% of transfused RBCs might be associated with a blood bank storage-associated reduction in deformability [5, 6]. This association would make deformability a valuable parameter, not only for evaluating blood bank quality but also for clarifying the relationship between various clinical conditions and RBC removal. Data on storage-associated RBC deformability obtained by ektacytometry, filtration, and micropipette aspiration have yielded conflicting results [3, 13–15, 37–39]. In contrast to these techniques, microfluidics analyzes deformation under conditions that mimic microcapillary flow conditions much more closely. The data we obtained with our microfluidic analysis strongly suggest that storage in blood bank conditions is not associated with alterations in deformability capacity of RBCs during their passage through the capillary system. Recent microfluidic data obtained with a comparable system also showed no significant effect of blood bank storage time on deformability, but a decrease in relaxation capacity [40]. Interestingly, the latter was associated with an increase in the circularity distribution width, indicating that the relaxation capacity is more strongly associated with changes to a more spherical cell shape than the deformation index. Also, the data of the glutaraldehyde-treated RBCs indicated that an increase in membrane stiffness leads to a decreased deformability in the narrow channels. It has been suggested that membrane loss by storage-associated vesiculation could be the main factor for a decreased deformability of red cells from transfusion units [6]. However, the lack of an effect of LPC-induced increase in surface-to-volume ratio on the deformation index or the relaxation index indicates that moderate changes in surface-to-volume ratio by itself do not induce any aberrant behavior in microcapillaries. This is in line with our observations on the normal behaviour of the most dense RBCs as obtained by separation on Percoll gradients. Nevertheless, more extensive changes in the surface-to-volume ratio could have an effect in conditions of more severe mechanical stress, as experienced by RBCs in the spleen [36].

Thus, all available data using a capillary-mimicking microfluidic approach suggest that the storage lesions, including the altered cell morphology, do not affect RBC behavior in the microcapillaries. However, an increased membrane rigidity in combination with an increased intracellular viscosity may be responsible for decreased capacity to pass through the spleen. These changes may be the effect of a vesiculation-induced increase in cellular hemoglobin concentration and a concomitant loss of functional membrane-cytoskeletal anchorage proteins during aging* in vivo* and* in vitro* [41, 42]. The loss of capacity to pass through the spleen may be detectable with the—more physically demanding—methods such as ektacytometry or the recently described spleen-mimicking device [43]. Our data strengthen the hypothesis that removal of transfused RBCs is mainly caused by the appearance of removal signals.

In conclusion, we show that microfluidics, when geared to flow conditions in capillaries, can yield substantial information on RBC deformability. We expect this approach to become a valuable tool for providing insights into RBC behavior in transfusion and hematological research. Our microfluidic analysis may be especially useful in the elucidation of the biologically relevant factors that are responsible for the large variation in the quality of RBC concentrates. In addition, our setup may be useful in evaluating the effects of disorders such as cardiovascular disease or systemic inflammation on red blood cell function.

## Supplementary Material

Supplemental Video 1: Deformation and relaxation of RBCs passing through microfluidic channels mimicking microcirculation in vivo. RBCs transit through the microchannels with an axisymmetric bullet-like shape. They relax back to their initial disc-like shape as soon as they exit into the relaxation zone.
